# Secondary bladder cancer after anticancer therapy for prostate cancer: reduced comorbidity after androgen-deprivation therapy

**DOI:** 10.18632/oncotarget.3817

**Published:** 2015-04-14

**Authors:** Masaki Shiota, Akira Yokomizo, Ario Takeuchi, Kenjiro Imada, Keijiro Kiyoshima, Junichi Inokuchi, Katsunori Tatsugami, Saiji Ohga, Katsumasa Nakamura, Hiroshi Honda, Seiji Naito

**Affiliations:** ^1^ Department of Urology, Graduate School of Medical Sciences, Kyushu University, Fukuoka, Japan; ^2^ Department of Clinical Radiology, Graduate School of Medical Sciences, Kyushu University, Fukuoka, Japan

**Keywords:** androgen-deprivation therapy, bladder cancer, prostate cancer, radical prostatectomy, radiotherapy

## Abstract

Radiotherapy for prostate cancer is associated with an increased incidence of secondary bladder cancer (BC). We investigated the incidence, clinicopathological characteristics, and prognosis of BC after radiotherapy, surgical therapy, and primary androgen-deprivation therapy (ADT) for prostate cancer. This study included 1,334 Japanese patients with prostate cancer treated with radiotherapy (n=631), surgical therapy (n=437), and primary ADT (n=266). During the median follow-up period of 51.2, 44.8, and 45.5 months, secondary BC occurred in 14 (2.2%), 5 (1.1%), and 0 (0%) of patients with prostate cancer treated with radiotherapy, surgical therapy, and primary ADT, respectively. The 10-year BC-free survival rate was 91.3% in the radiotherapy group, 97.4% in the surgical therapy group, and 100% in the primary ADT group. The rates of intravesical recurrence, progression to muscle-invasive BC, and BC-specific death might be higher in secondary BC after radiotherapy compared with after surgical therapy. There was a significant difference in the incidence of secondary BC among different therapeutic modalities for prostate cancer in Japanese men, indicating significantly lower comorbidity rates of secondary BC after primary ADT for prostate cancer compared with radiotherapy.

## INTRODUCTION

Prostate cancer is one of the most common cancers among men of developed countries. In the United States, it is estimated that about 233,000 men will develop, and about 29,480 men will die, from prostate cancer in 2014 [1]. In Japan, the morbidity rate of prostate cancer has been increasing owing to various reasons, including the growing prevalence of a Western-style diet and lifestyle, and an increase in the aging population as well as prostate specific antigen (PSA) screening [2].

For prostate cancer, several therapeutic modalities are available and apply to patients with prostate cancer according to various clinicopathological parameters. Standard treatment options for localized prostate cancer include: radical prostatectomy (RP) by open RP, laparoscopic RP, or robot-assisted laparoscopic RP; and prostate radiotherapy by external beam radiotherapy (EBRT) and/or brachytherapy (BT). Additionally, salvage radiotherapy is a common therapeutic option for biochemical recurrence after RP along with salvage androgen-deprivation therapy (ADT). In contrast, ADT inhibiting androgen receptor (AR) signaling by either surgical or medical castration and/or anti-androgen agents is the gold standard for advanced or metastatic prostate cancer. Among these therapeutic modalities, physicians and patients can choose the best single option or combination. As patients with prostate cancer are expected to live longer compared with those with other cancers, late-phase adverse events are more meaningful and critical. There are increasing numbers of prostate cancer survivors with severe, long-lasting adverse events resulting from prostate cancer therapy. However, although numerous studies have reported the oncological outcomes using these modalities, reports on adverse events, especially late-phase events, are relatively limited.

Bladder cancer (BC) is one of the most common urogenital cancers, usually characterized as urothelial carcinoma. In the United States, it is estimated that about 74,690 people will develop BC and about 15,580 people will die from BC in 2014 [1]. Previously, a relationship between prostate cancer and BC has been suggested. Etiologically, the high incidence of double primary cancer (prostate cancer and BC) has been reported [3], which is supported by the biological findings that common molecular pathways, such as androgen receptor (AR) signaling, contributes to the carcinogenesis and tumorigenesis of prostate cancer as well as BC [4, 5]. However, diagnostic bias and other factors, such as smoking and metabolic syndrome, are confounding variables of this relationship [6]. In reports from the Chernobyl Nuclear Power Plant accident in Ukraine, ionizing radiation was shown to induce DNA damage and gene mutations leading to urothelial dysplasia [7, 8]. In addition to mutagenesis of epithelial cells caused by irradiation, more recently, the irradiated microenvironment has also been recognized to promote the tumorigenesis through tumor growth factor (TGF)-β signaling [9]. It has been reported that the incidence of secondary BC, which is one of most critical adverse events, increases after radiotherapy for prostate cancer, likely owing to mutagenicity by radiation [10].

However, the hypothesis that radiotherapy may increase secondary BC is vague because most studies lack a comparable approach or rely on community-based registry databases that have strong statistical power but less reliability and data accuracy. Moreover, reports on secondary BC have been derived from mostly North American data. Additionally, the comorbid rate of secondary BC when treated with ADT for prostate cancer is unknown. Therefore, in this study, the incidence of BC comorbid with prostate cancer was investigated using single institution data according to prostate anticancer treatment, including radiotherapy, surgical therapy, and primary ADT. In addition, the clinicopathological characteristics and prognosis of secondary BC among therapeutical modalities for prostate cancer were evaluated.

## RESULTS

This study enrolled a total of 1,334 patients, whose clinical and pathological characteristics are shown in Table 1. The median age of patients was 71, 65, and 74 years and the median PSA at diagnosis was 9.0, 7.7, and 48.0 ng/mL among radiotherapy, surgical therapy, and ADT groups, respectively. The Gleason scores of biopsy specimens from 119 (18.9%) patients, 64 (14.6%) patients, and 134 (50.4%) patients treated with radiotherapy, surgical therapy, or ADT, respectively, were >7. Among patients treated with ADT, 102 (34.7%) and 139 (47.3%) men had regional lymph node and/or distant metastases, respectively, while only a few or none of the patients treated with radiotherapy or surgical therapy had lymph node or distant metastases, respectively. Thus, compared with patients treated with radiotherapy and surgical therapy, patients treated with ADT were older and had worse characteristics, such as higher PSA values at diagnosis, higher Gleason scores, and more progressive TNM-stage, indicating an appropriate indication for primary ADT to treat advanced prostate cancer in this cohort.

**Table 1:** Patient characteristics

Variable	Radiotherapy (n=631)	Surgical therapy (n=437)	ADT (n=266)	*p*-value
Median age, years (IQR)	70 (65-74)	65 (60-69)	74 (69-78)	<0.0001
Median PSA at diagnosis, ng/ml (IQR)	9.0 (6.3-15.5)	7.6 (5.5-11.7)	49.7 (13.6-207.6)	<0.0001
NA, n (%)	3 (0.5%)	1 (0.2%)	5 (1.9%)	
Biopsy Gleason score, n (%)				
<7	258 (40.9%)	170 (38.9%)	39 (14.7%)	
7	238 (37.7%)	188 (43.0%)	75 (28.2%)	
≥8	119 (18.9%)	64 (14.6%)	134 (50.4%)	<0.0001
NA	16 (2.5%)	15 (3.4%)	18 (6.8%)	
Clinical T-stage, n (%)				
T1	322 (51.0%)	247 (56.5%)	40 (15.0%)	
T2	187 (29.7%)	152 (34.8%)	54 (20.3%)	
T3	100 (15.8%)	9 (2.1%)	111 (41.7%)	
T4	11 (1.7%)	0 (0.0%)	47 (17.7%)	<0.0001
NA	11 (1.7%)	29 (6.6%)	14 (5.3%)	
Clinical N-stage, n (%)				
N0	618 (97.9%)	437 (100%)	161 (60.5%)	
N1	10 (1.6%)	0 (0.0%)	97 (36.5%)	<0.0001
NA	3 (0.5%)	0 (0.0%)	8 (3.0%)	
Clinical M-stage, n (%)				
M0	624 (98.9%)	437 (100%)	129 (48.5%)	
M1	4 (0.6%)	0 (0.0%)	130 (48.9%)	<0.0001
NA	3 (0.5%)	0 (0.0%)	7 (2.6%)	

IQR, Interquartile range; NA, not available.

**Figure 1:**
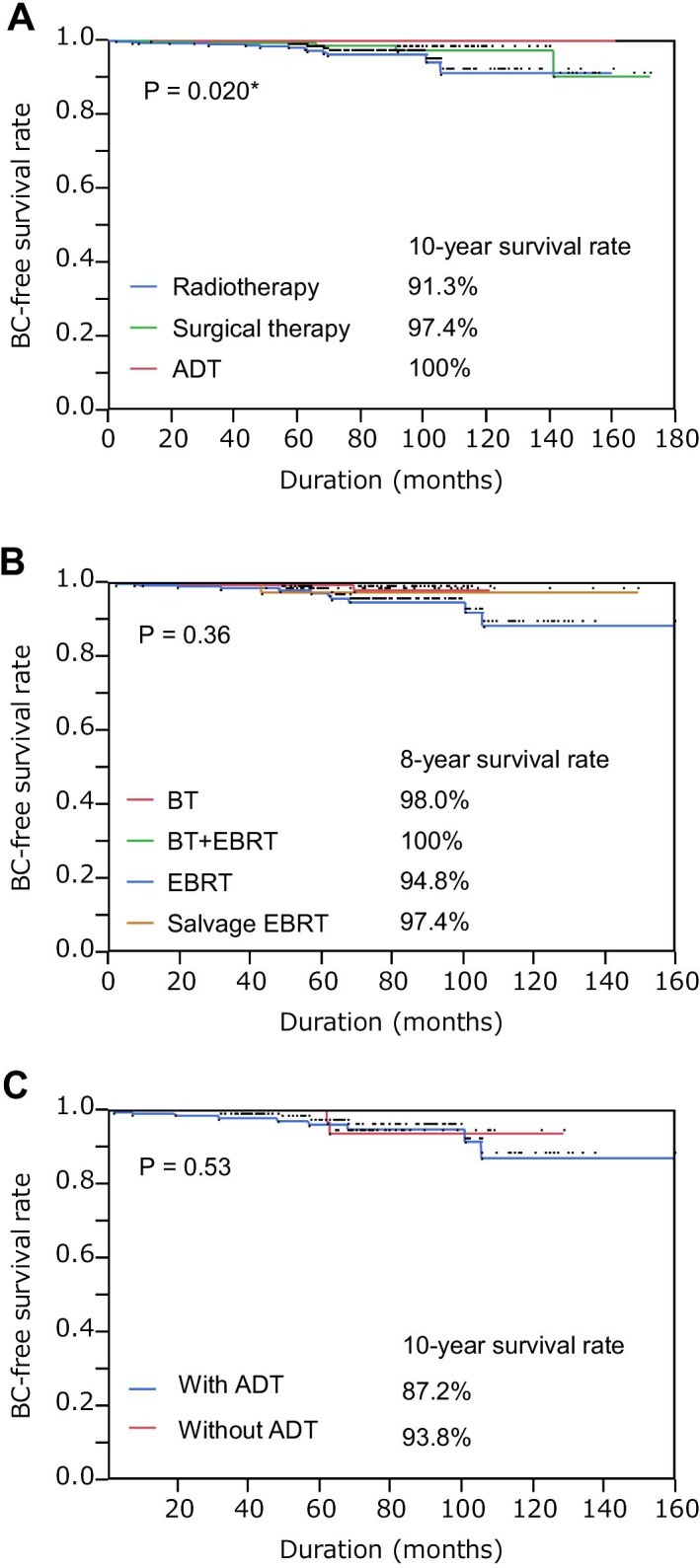
Secondary bladder cancer-free survival rates in patients with prostate cancer treated with the indicated modalities (**A**) Secondary bladder cancer (BC)-free survival rate in patients treated with radiotherapy, surgical therapy, or primary androgen deprivation therapy (ADT); *P < 0.05. (**B**) Secondary BC-free survival rate in patients treated with brachytherapy (BT), external beam radiotherapy (EBRT), BT + EBRT, or salvage EBRT. (**C**) Secondary BC-free survival rate in patients treated with EBRT with or without ADT.

The cancer comorbid with prostate cancer was categorically divided into preceding, concurrent, and subsequent cancer according to the timing of diagnosis; cancer 1 year before or after prostate cancer diagnosing was defined as preceding or subsequent cancer, respectively, while cancer diagnosed within 1 year before or after diagnosis with prostate cancer was defined as concurrent cancer. As shown in Table 2, among these 1,334 patients, 49 cases of comorbid BC were diagnosed in 10 (0.7%), 20 (1.5%), and 19 (1.4%) men with preceding, concurrent, and subsequent BC, respectively. With regard to therapeutic modalities, 23 men (3.6%), 11 men (2.5%), and 15 men (5.6%) among patients treated with radiotherapy, surgical therapy, and primary ADT, were diagnosed with BC, respectively. Focusing on secondary BC, after excluding preceding and concurrent BC, during the median follow-up period of 51.2 (IQR, 27.4–75.9) months, 14 men (2.2%) among patients treated with radiotherapy were diagnosed with secondary BC with a median latency of 52.3 (IQR, 16.6–68.0) months. Conversely, during the median follow-up period of 44.8 (IQR, 24.4–77.7) and 45.5 (IQR, 26.1–71.3) months, five (1.1%) men or no men among patients treated with surgical treatment or ADT were diagnosed with secondary BC with a median latency of 65.8 (IQR, 19.8–116.0) months, respectively. Accordingly, as shown in Fig. 1A, the 10-year BC-free survival rate was 91.3%, 97.4%, and 100% among patients treated with radiotherapy, surgical therapy, and ADT, respectively (*P* = 0.020; radiotherapy vs. surgical therapy, *P* = 0.11; radiotherapy vs. ADT, *P* = 0.016: surgical therapy vs. ADT, *P* = 0.090). Thus, the incidence of secondary BC was higher among patients treated with radiotherapy although statistical significance was not reached, while, surprisingly, the incidence of secondary BC was significantly lower among patients treated with ADT. Because docetaxel chemotherapy might affect the occurrence of secondary BC, the comorbid rate of secondary BC was only compared among patients without docetaxel chemotherapy. Although docetaxel chemotherapy was administered to one (0.2%), two (0.5%), and 31 men (11.7%) in radiotherapy, surgical therapy, and ADT groups, respectively, the conclusion was similar even when these cases were excluded (data not shown). According to therapeutic radiotherapy modality, during the median follow-up period of 54.7 (IQR, 30.5–73.8), 72.3 (IQR, 57.5–88.6), 45.2 (IQR, 23.1–74.1), and 52.1 (IQR, 31.8–83.5) months, 8-year BC-free survival rates were 98.0%, 100%, 94.8%, and 97.4% in BT, BT + EBRT, EBRT, and salvage EBRT groups, respectively (Fig. 1B). Thus, the incidence of secondary BC was higher in the EBRT group compared with BT, BT + EBRT, and salvage EBRT groups, although statistical significance was not reached (*P* = 0.357). Moreover, because neoadjuvant/adjuvant ADT was used with EBRT, the comorbid rate of secondary BC was examined between EBRT with ADT (n = 239) or without ADT (n = 102), which showed similar BC-free survival rates (Fig. 1C).

**Table 2:** Incidence of BC and the temporal relationship with prostate cancer diagnosis

Therapeutical modality	n	Preceding (n=10)	Concurrent (n=20)	Subsequent (n=19)	Total (n=49)
Radiotherapy	631	4 (0.6%)	5 (0.8%)	14 (2.2%)	23 (3.6%)
BT	206	1 (0.5%)	0 (0.0%)	2 (1.0%)	3 (1.5%)
BT+EBRT	13	0 (0.0%)	0 (0.0%)	0 (0.0%)	0 (0.0%)
EBRT	348	3 (0.9%)	5 (1.4%)	11 (3.2%)	19 (5.5%)
Salvage EBRT	64	0 (0.0%)	0 (0.0%)	1 (1.6%)	1 (1.6%)
Surgical therapy (without radiotherapy)	437	2 (0.5%)	4 (0.9%)	5 (1.1%)	11 (2.5%)
Open RP	216	1 (0.5%)	1 (0.5%)	3 (1.4%)	5 (2.3%)
Laparoscopic RP	71	0 (0.0%)	0 (0.0%)	1 (1.4%)	1 (1.4%)
Robot-assisted laparoscopic RP	222	0 (0.0%)	0 (0.0%)	1 (0.5%)	1 (0.5%)
Radical cystectomy	4	1 (25.0%)	3 (75.0%)	0 (0.0%)	4 (100%)
ADT (without radiotherapy)	266	4 (1.5%)	11 (4.1%)	0 (0.0%)	15 (5.6%)
Castration+anti-androgen	193	1 (0.5%)	9 (4.7%)	0 (0.0%)	10 (5.2%)
Castration	62	2 (3.2%)	1 (1.6%)	0 (0.0%)	3 (4.8%)
Anti-androgen	11	1 (9.1%)	1 (9.1%)	0 (0.0%)	2 (18.2%)

Finally, the clinicopathological characteristics of secondary BC among patients treated with radiotherapy or surgical therapy was investigated. Among the clinicopathological parameters listed in Table 3, tumor site, tumor number, tumor size, carcinoma in situ (CIS), histological grade, and local stage were comparable between patients treated with radiotherapy and surgical therapy, although urothelial carcinoma in prostatic urethra characteristically occurred in two cases treated with radiotherapy. However, as shown in Supplementary Fig. 1 and Table 3, among patients with non-MIBC (n = 15), intravesical recurrence-free survival rate (*P* = 0.32), progression to muscle-invasive BC-free survival rate (*P* = 0.40), and BC-specific survival rate (*P* = 0.42) were inferior in those treated with radiotherapy compared with patients treated with surgical therapy, although statistical significance was not reached. Notably, three (0.5%) men treated with radiotherapy for prostate cancer died from BC; however, nine (1.4%) patients in the radiotherapy series died from prostate cancer.

**Table 3:** Cliniopathological characteristics of secondary BC

Clinicopathological parameters	Radiotherapy	Surgical therapy
Initial symptom		
Hematuria	9	5
Scrotal swelling	1	0
Incidental	4	0
Tumor site		
Prostate	2	0
Neck	0	0
Trigone	2	1
Lateral wall	6	3
Dome	0	1
Posterior wall	5	1
Anterior wall	1	0
Tumor number		
1	7	1
2~7	2	3
≥8	2	1
Not applicable	3	0
Tumor size		
<3cm	10	4
≥3cm	1	0
Not applicable	3	1
CIS		
Absent	11	4
Present	3	1
Histological type		
Urothelial carcinoma	14	4
Squamous cell carcinoma	0	1
Grade		
Low grade	6	0
High grade	8	4
Not applicable	0	1
Local stage		
Non-MIBC	11	4
MIBC	3	1
Metastasis at primary diagnosis		
Absent	12	5
Present	2	0
Accompanied upper tract urothelial carcinoma		
Absent	12	4
Present	2	1
Intravesical instillation for NMIBC		
Anticancer agent	4	2
Bacillus Calmette-Guérin (BCG)	4	1
Single instillation alone	3	1
Intravesical recurrence		
Absent	8	4
Present	3	0
Progression to MIBC		
Absent	9	4
Present	2	0
Therapeutic for non-metastatic MIBC		
Radical cystectomy	1	1
Chemotherapy	1	0
Best supportive care	1	0
Therapeutic for metastatic BC		
Chemotherapy	1	0
Best supportive care	1	0
BC-specific death		
Absent	12	5
Present	3	0

## DISCUSSION

It has been suggested that radiotherapy for prostate cancer can induce secondary BC. Using community-based registry databases, several studies have shown an increased incidence of secondary BC after radiotherapy for prostate cancer. Studies using Surveillance, Epidemiology, and End Results (SEER) database-which includes hundreds of thousands of prostate cancer cases-indicate that BC risk increases after radiotherapy for prostate cancer compared with the non-radiotherapy group, such as surgery and no treatment [11-16]. Similarly, Boorjian et al. reported an increased risk of BC after radiotherapy compared with surgical treatment for prostate cancer using the Cancer of the Prostate Strategic Urologic Research Endeavor (CaPSURE) database including about 10,000 cases of prostate cancer [17]. Additionally, Pawlish et al., using the Detroit SEER database [18], showed a significant increase in secondary BC risk following radiotherapy. However, in contrast, several studies using smaller cohorts failed to show a significant increased risk of secondary BC. For instance, Chrouser et al. at the Mayo Clinic [19], Movsas et al. at the Fox Chase Cancer Center [20], and Pickles et al. using the British Columbia Tumor Registry [21] reported no risk change in incidence after radiotherapy. Consistent with these controversial previous studies, the present study showed only a marginal increased risk of secondary BC after radiotherapy for prostate cancer when compared with surgical therapy probably because of the small sample size, but significant when compared with primary ADT.

Previously, a differential effect on secondary BC by distinct radiotherapy modalities has been proposed. Liauw et al. reported secondary BC after BT alone or combined with EBRT at the Seattle Prostate Institute, showing a decreased risk of secondary BC after BT alone compared with BT combined with EBRT [22]. Moreover, Moon et al. showed a similar result using data from the SEER registry in that BT did not affect the incidence of secondary BC compared with EBRT [13]. Based on these findings, it is possible that decreased irradiation dose to the bladder may contribute to reducing the risk of secondary BC. In the present study, we showed a decreased risk of secondary BC after BT compared with EBRT, although statistical significance was not reached likely because of the small sample size.

Surgery for prostate cancer reportedly does not increase secondary BC risk, as reported by studies using the SEER database [12, 15, 16]. With regard to ADT, there are no reports investigating secondary BC. In this study, unexpectedly and surprisingly, the cohort treated with primary ADT was at a significantly lower risk of secondary BC compared with radiotherapy, irrespective of older age among the primary ADT series, which is an established risk factor for secondary BC [16]. To the best of our knowledge, this is the first report of the comorbidity rate of secondary BC after ADT, suggesting that ADT might reduce the risk comorbid with secondary BC by suppressing urothelial carcinogenesis and tumorigenesis. Previously, AR signaling has been suggested to be involved in the carcinogenesis and tumorigenesis of the prostate as well as the urothelium [4, 23]. It is consistently reported that AR signaling promotes DNA breaks and chromosomal rearrangement, leading to the emergence of oncogenic fusion-genes [24, 25]. We have also recently shown that androgen depletion and AR knockdown suppressed cell proliferation in BC expressing ARs [5]. Taken together, these biological findings may account for the lower comorbidity rate of secondary BC among men treated with ADT in this study. Furthermore, Izumi et al. has recently reported that ADT for prostate cancer concurrent with non-MIBC can reduce intravesical recurrence rate of BC, supporting the results of this study from a clinical perspective [26]. Conversely, the comorbid rate of secondary BC after radiotherapy was similar between patients who did or did not undergo ADT, suggesting that ADT might affect usual bladder carcinogenesis, but not irradiation-induced bladder carcinogenesis, or the relatively short period of neoadjuvant/adjuvant ADT was not sufficient to prevent bladder carcinogenesis.

Radiotherapy, and especially EBRT for prostate cancer, may increase the risk of secondary BC. However, the clinicopathological characteristics of secondary BC remain unclear. In this study, several characteristics of secondary BC after radiotherapy have been reported, which includes a higher possibility of urothelial tumor in the prostate, intravesical recurrence, progression to MIBC, and BC-specific death, although statistical significance was not reached, likely because of the small sample size. Similarly, Cherouser et al. reported frequent recurrence and progression of secondary BC after radiotherapy for prostate cancer [19]. Moreover, Bostrom et al. showed worse prognosis comparing data from patients after radiotherapy for prostate cancer compared with a matched control group [27]. Additionally, Abern et al. reported a higher incidence of non-urothelial carcinoma, trigonal-located cancer, CIS, and BC-specific death using SEER database [28]. In line with these studies, higher BC grade and stage after radiotherapy has been reported from the Memorial Sloan Kettering Cancer Center Tumor Registry [29, 30] and the University of Miami [27, 31]. Also, in *in vitro* experiments, irradiation has been shown to promote the progression from low-grade urothelial cancer to high-grade cancer [32]. Thus, secondary BC might occur in a region near the prostate, and have high-risk properties of recurrence and lethal progression, as shown in this study. Conversely, the worse prognosis may be derived from the pre-history of radiotherapy to prostate, which limited the subsequent surgical therapy for BC, such as radical cystectomy.

The present study had several limitations. For instance, the study design was retrospective, the sample size was relatively small, and the follow-up period was relatively short. Despite these limitations, this study showed a significant difference in the incidence of secondary BC among therapeutic modalities for prostate cancer in a single institution. However, detection bias by symptoms such as gross-hematuria and bladder irritation, which are often caused by radiotherapy, and short life expectancy due to patient characteristics in the ADT group may have affected the results. Selection bias of therapy for prostate cancer may also have affected the results. Radiation-induced malignancies usually occur more than 4–5 years after therapy, but our study included six cases with secondary BC that occurred within 4 years. In addition, the lack of data pertaining to smoking status is another limitation of this study. Conversely, this study included a cohort from the urological department from only one institution, assuring high integrity of the data, in contrast to data from community-based databases.

## CONCLUSION

This study showed a significant difference in the incidence and possible differences in clinicopathological characteristics and prognosis of secondary BC among different therapeutic modalities for prostate cancer in Japanese men treated in a single institution; we show a significantly lower comorbidity rate of secondary BC after ADT for prostate cancer. Thus, this study has opened up the new possibility that ADT might clinically suppress BC occurrence. Accordingly, further exploration of the aforementioned possibility is warranted.

## MATERIALS AND METHODS

This study enrolled patients with prostate cancer treated by radiotherapy, surgical therapy, or primary ADT at Kyushu University Hospital (Fukuoka, Japan) from 2000 to 2012. This study was approved by the Kyushu University Hospital institutional review board. All patients were histopathologically diagnosed with adenocarcinoma of the prostate. Patients with less than 1 year follow-up after therapy were excluded. Clinical staging was determined in accordance with the unified TNM criteria based on the results of a digital rectal examination, transrectal ultrasound, computed tomography, magnetic resonance imaging, and bone scan [33]. Most patients also underwent cystoscopy to determine tumor extension and to rule out comorbid BC. Patients in the radiotherapy series were treated by brachytherapy (BT), EBRT, BT + EBRT, or salvage EBRT with or without neoadjuvant/adjuvant ADT (more than 3 months) by surgical castration or medical castration using a luteinizing hormone-releasing hormone agonist (goserelin acetate or leuprorelin acetate) and/or an antiandrogen agent (bicalutamide, flutamide, or chlormadinone acetate). BT [34], EBRT [35, 36], and salvage EBRT [37] were performed as described previously. Briefly, BT was performed using permanent implantation of ^125^I. For BT + EBRT, 45 Gy of EBRT to prostate was added after BT. EBRT was performed with a median 72 (interquartile range [IQR], 70–72) Gy to the prostate only (n = 294, 84.5%) or the prostate and pelvis (n = 48, 13.8%) by conformal (n = 312, 89.7%) or intensity-modulated radiotherapy (n = 30, 8.6%). Salvage EBRT was conducted with a median 66 (IQR, 64.8–66) Gy to the prostatic bed (n = 58, 90.6%) or pelvis (n = 6, 9.4%) by conformal (n = 63, 98.4%) or intensity-modulated radiotherapy (n = 1, 1.6%). Patients in the surgical therapy series were treated by open RP, laparoscopic RP, robot-assisted laparoscopic LP, or radical cystectomy with or without ADT [6]. Patients who received salvage radiotherapy were excluded from the surgical therapy series. Patients in the ADT series were primarily treated with ADT because of metastasized disease, advanced age, severe comorbidity, low performance status or patient preference. Patients who received surgical therapy or radiotherapy for prostate cancer were excluded from the ADT series. Patients were followed up during or after treatment for prostate cancer at intervals of about 3–6 months with medical interview, urinalysis, and blood test. If BC incidence was suspected, further examinations, such as urine cytology and cystoscopy, were performed. The time to development of secondary cancer was defined as the duration from the date of principal therapeutic start for prostate cancer to the date of pathological diagnosis of secondary cancer.

Primary BC, but not recurrent BC, was included in this study. All comorbid BC were histologically confirmed. Pathological evaluation of BC was performed according to 2004 World Health Organization (WHO) grading [38]. In this study, urothelial carcinoma in prostatic urethra was included in the BC cohort. Clinical staging of BC was determined in accordance with the unified TNM criteria based on the results of a bimanual examination, transurethral resection, computed tomography, magnetic resonance imaging, and bone scan [33]. The time to intravesical recurrence, progression to muscle-invasive bladder cancer (MIBC), or BC-specific death was defined as the duration from the date of pathological diagnosis of secondary cancer to the date of intravesical recurrence, progression to MIBC, or BC-specific death, respectively.

All statistical analyses were performed using JMP9 software (SAS Institute, Cary, NC, USA). The survival rates were determined using the Kaplan–Meier method and the log-rank statistic was used to compare survival duration across groups. Comparisons between groups were analyzed by Wilcoxon or Pearson tests. *P*-values < 0.05 were considered significant.

## SUPPLEMENTARY MATERIAL AND FIGURE


