# Supplementary material for: Secondary bladder cancer after anticancer therapy for prostate cancer: reduced comorbidity after androgen-deprivation therapy
Source: Oncotarget. 2015 Apr 14;6(16):14710–9. doi: 10.18632/oncotarget.3817 (PMC4546499; doi:10.18632/oncotarget.3817)
Supplement: Supplementary file 1 [file oncotarget-06-14710-s001.pdf]

# Secondary bladder cancer after anticancer therapy for prostate cancer: reduced comorbidity after androgen-deprivation therapy

**Supplementary Material**

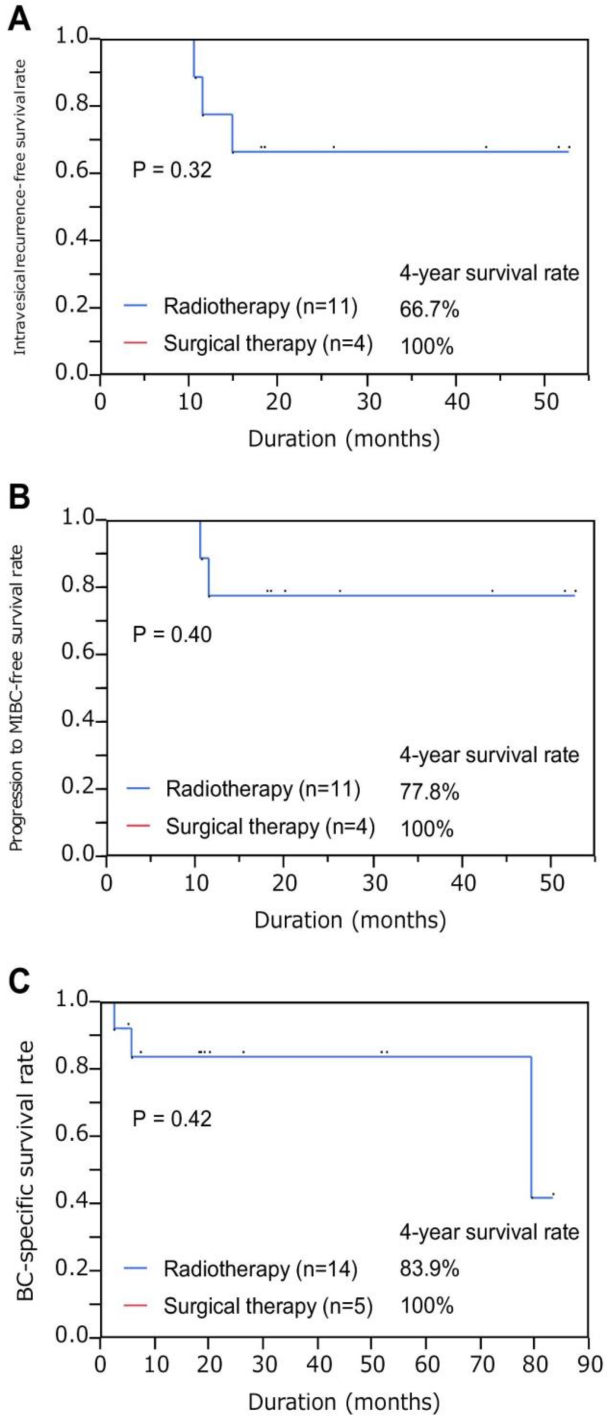


Supplementary Figure1
